# Vasohibin 2 Decreases the Cisplatin Sensitivity of Hepatocarcinoma Cell Line by Downregulating p53

**DOI:** 10.1371/journal.pone.0090358

**Published:** 2014-03-04

**Authors:** Zhanjun Li, Min Tu, Bei Han, Yuqing Gu, Xiaofeng Xue, Jie Sun, Qianqian Ge, Yi Miao, Zhuyin Qian, Wentao Gao

**Affiliations:** 1 Laboratory of General Surgery, the First Affiliated Hospital of Nanjing Medical University, Nanjing, China; 2 Department of General Surgery, the First Affiliated Hospital of Nanjing Medical University, Nanjing, China; 3 Department of Endocrinology, Nanjing Children's Hospital Affiliated to Nanjing Medical University, Nanjing, China; 4 Department of General Surgery, Taicang City First People's Hospital, Suzhou, China; 5 Department of General Surgery, the First Affiliated Hospital of Suzhou University, Suzhou, China; 6 Department of General Surgery, Fuyang People's Hospital, Fuyang, China; University of Navarra School of Medicine and Center for Applied Medical Research (CIMA), Spain

## Abstract

Hepatocellular carcinoma (HCC) is a prevalent problem worldwide. Chemotherapy, especially cisplatin (CDDP)-based systemic chemotherapy, is the best option for advanced liver cancer. However, CDDP resistance is becoming common and hindering the clinical application of CDDP. Meanwhile, no consensus has been reached regarding the chemotherapeutic use of vasohibin 2 (VASH2), which promotes the angiogenesis and proliferation of cancer cells. In this work, a tissue microarray was used to observe VASH2 and its possible role in cancer treatment. Results showed that VASH2 was highly expressed in HCC tissues and was significantly correlated with cancer differentiation. To further investigate the efficacy and mechanism of the combination of VASH2 with anti-cancer drugs in liver cancer cells, we stably built VASH2 overexpression and knockdown cell lines. We found that VASH2 can influence the CDDP sensitivity and that the cell overexpression of VASH2 had a higher cell viability and lower apoptosis rate after CDDP exposure. We also observed that VASH2 overexpression downregulated wild-type p53, as well as suppressed the expression of the pro-apoptotic protein BCL2-associated X protein (Bax) and cleaved caspase-3 (CC-3) after treatment by CDDP. Conversely, the knockdown of VASH2 significantly inhibited these effects. In an *in vivo* chemosensitivity study, nude mice were subcutaneously injected with tumor cells and received CDDP treatment through intraperitoneal administration every 3 days. We found that VASH2 knockdown markedly limited the tumor growth and enhanced the CDDP toxicity and apoptosis of tumor cells. Western blot analysis revealed that tumor cells with downregulated VASH2 had a higher expression of wild-type p53, Bax, and CC-3 than control cells. Overall, our results indicated the novel roles of VASH2 in the chemoresistance of hepatocarcinoma cells to CDDP and suggested that VASH2 may be a promising anticancer target.

## Introduction

Vasohibin 2 (VASH2) belongs to the VASH family along with vasohibin 1 (VASH1). VASH2, which was first described by Shibuya et al. [1], is located on chromosome 1q32.3 and composed of 355 amino acid residues. The overall homology between human VASH1 and VASH2 is 52.5% at the amino acid level [2]. VASH1 is restricted to endothelial cells (ECs) and induced by the potent angiogenic factors VEGF and FGF-2 [3], [4]. Many studies have reported that VASH1 is involved in angiogenesis in various solid tumors and that exogenous VASH1 significantly blocks sprouting angiogenesis by tumors [5]–[7]. In contrast to VASH1, VASH2 not only promotes angiogenesis, but also highly expresses in HCC cells and tissues and promotes HCC cell proliferation and tumor growth [8]–[10]. These results indicate that the function of VASH2 is beyond angiogenesis promotion, *i.e.*, it plays a significant role in other aspects of tumor metabolism.

To further characterize the significance of VASH2, we successfully produced anti-human VASH2 polyclonal antibody [11]. In addition to finding the expression level of VASH2 higher in HCC tumors than in normal livers, we also observed that it gradually increased with decreased degree of tumor differentiation. The curative effect of chemotherapeutic drug is also reportedly correlated with the grade of tumor differentiation [12]–[18], and the efficacy of an anti-cancer drug can be enhanced with the differentiation of cancer cells facilitated by cell-differentiation inducers [19]. Based on these findings, we hypothesize that VASH2 maybe involved in the mechanisms of cancer chemotherapy.

CDDP is extensively used as a chemotherapeutic agent for the treatment of HCC and other human solid tumors [20].To confirm whether VASH2 participate in the treatment of CDDP, We then stably built VASH2 overexpression and knockdown cell lines. Both *in vivo* and *in vitro* analyses demonstrated that VASH2 conferred HepG2 and SMMC7721 cells with chemical resistance to CDDP. But, how VASH2 influences the sensitivity of CDDP? Given that CDDP induces cell death by forming various adducts with DNA and activates the p53 pathway [21]–[24]. So, we determined the expression level of p53 after up or down regulating VASH2 and could not find the change of p53 in mRNA level, unfortunately. But, to our surprised, upregulation of VASH2 distinctly decreased the expression of p53 in protein level. Next, we also discovered that VASH2 could suppress the expression of the pro-apoptotic protein Bax and cleaved caspase-3 (CC-3). Therefore, VASH2 maybe influence the sensitivity of CDDP by downregulating p53 and inhibiting apoptosis.

## Materials and Methods

### Ethics statement

All animal studies were reviewed and approved by the Ethics Committee of Nanjing Medical University in accordance with the established standards of the humane handling of research animals.

### Tissue microarray

HCC sample tissue microarray (LV1021) and normal liver tissue microarray (FDA999b) used for immunohistology analysis (IHC) were purchased from Alenabio (Xi'an, China). Histopathological grading and clinical TNM classification strictly followed the Edmondson−Steiner pathological grading method [25] and TNM clinical staging method [26], respectively. VASH2 antibody was used as previously described [11]. The staining pattern of VASH2 was classified in a subjective spectrum of 0 to +++ as follows: 0, negative expression in tumor tissue; +, weak staining; ++, moderate staining; and +++, strong staining. For each staining level, the percentage of cells with a specific score was visually estimated. When <10% of the cells were positively stained, the section was classified as negative. Positive sections were further divided into weakly positive (10% to 30%), moderately positive (30% to 50%), and strongly positive (more than 50%). The tissue microarray chips were observed under 20× magnification.

### Cell culture and establishment of VASH2-expressing tumor cell clones

HepG2 cell line was obtained from the American Type Tissue Culture Collection (Manassas, VA, USA). SMMC-7721 cell line was purchased from the China Center for Type Culture Collection (CCTCC). Tumor cell clones overexpressing or downexpressing VASH2 were successfully established as previously described [8]. Cells were cultured in Dulbecco's modified Eagle's medium (DMEM) containing 10% fetal bovine serum (Wisent, Canada), 100 mg/ml penicillin, and 100 mg/ml streptomycin (Thermo Scientific Hyclone, USA) at 37°C with 5% CO_2_.

### Quantitative RT-PCR

Total RNA was extracted from cells and tissues using RNAiso Plus reagent (Takara, Dalian, China), and cDNA was synthesized using Primescript RT Reagent (Takara). Quantitative RT-PCR was performed on a StepOnePlus Real-Time–PCR System (Applied Biosystems, Carlsbad, CA, USA) according to the manufacturer's instructions. The PCR conditions consisted of an initial denaturation step at 95°C for 10 min, followed by 40 cycles of 15 s at 95°C and 1 min at 60°C. Finally, a melting curve profile was set at 95°C (15 s), 60°C (15 s), and 95°C (15 s). Each mRNA level was measured as a fluorescent signal corrected according to the signal for β-actin. The primer pairs used were as follows: human VASH2 forward, 5′CTCTTCCAGCCTTCCTTCCT3′; reverse, 5′AGCACTGTGTTGGCGTACAG3′; human β-actin forward, 5′GGGAGAAATGGTGGGCG3′; reverse, 5′GCCAGTCTGGGATCGTCATC3′. Relative quantification was calculated by the ΔΔCt method and normalized based on β-actin.

### Cell proliferation and toxicity tests

Cytotoxicity was determined using the Cell Counting Kit-8 (CCK-8) method (Dojindo, CK04, Japan). Five groups of cells in logarithmic growth were kept in DMEM supplemented with 10% FBS. The confluent monolayers were trypsinized, washed with DMEM, and transferred to 96-well microtiter plates (4×10^4^ cells/well). After 16–18 h of pre-incubation, the medium was removed and CDDP was incubated for 48 h (37°C, 5% CO_2_). In this test, CDDP (Sigma–Aldrich, St. Louis, MO, USA) was serially diluted in different concentrations (1000 → 0.1 µg/ml) [27], [28]. Each group was seeded in five duplicates. Colorimetric reaction was developed after incubation with CCK-8 (37°C, 5% CO_2_, 1 h) following the manufacturer's instructions. Finally, absorbance was measured using a microtiter plate reader (Tecan, Salzburg, Austria) at 450 nm wavelength. The cell viability of each group was calculated by averaging the optical density.

### Cell apoptosis analysis

Cell apoptosis was assessed by flow cytometry (Becton Dickinson, San Jose, CA, USA). Cells were treated with CDDP (10 or 20 µg/ml) for 48 h. Cell pellets were collected, washed with PBS, and suspended in 100 µl of 1× binding buffer. The cell pellets were then stained with 5 µl of Phycoerythrin (PE)−Annexin-V and 5 µl of 7-AAD at room temperature for 15 min in the dark. The stained cells were immediately analyzed by flow cytometry. Data were analyzed with FlowJo software.

### *In vivo* chemosensitivity assay

Four-week-old female BALB/c nude mice were purchased from The Model Animal Research Center of Nanjing University, Nanjing, China. For *in vivo* chemosensitivity analyses, mice were randomly separated to four groups (9 mice per group). HepG2-EGFP, HepG2-VASH2, HepG2-shcont, and HepG2-shVASH2 cells were subcutaneously injected into the flanks of the mice (10^6^ cells/100 µl per flank). After injection with liver cancer cells for 9 days when the tumor volume has not statistically significant, each group of the mice were further divided into two subgroups, CDDP(−) and CDDP(+). The mice in CDDP(+) group began receiving CDDP (10 mg/kg) (Nanjing, China) intraperitoneally every 3 days [29],while CDDP(−) group was not received this treatment. Tumor volume was calculated using the formula (*W* ^2^×*L*)/2, where *L* is the length of the tumor and *W* is the width of the tumor. Bidimensional tumor measurements were conducted with calipers every 3 days. After six 3-day cycles of treatment, all nude mice were sacrificed and tumors were excised for further study.

### Terminal deoxynucleotidyl transferase dUTP nick end labeling (TUNEL) assay

All tumors were collected from the sacrificed mice after 27 days. The tumors were dried and paraffin embedded, and serial sections were prepared on glass slides coated with poly-lysine. One section was randomly selected from each serial section so that each treatment group was represented by four tumor samples dyed for TUNEL assay. The sections were observed under 40× magnification. Five visual fields were randomly selected from each section, from which the total cell number and number of apoptotic cells were counted to calculate the proportion of apoptotic cells. Cells showing amethyst granules in the cell nucleus were deemed positive for apoptosis. The apoptotic rate of cancer cells was calculated as apoptotic cells/total cells × 100%.

### Western blot analysis

Cell and xenograft tumor lysates were prepared by extracting protein with radio immunoprecipitation assay buffer which is a rapid cell and tissue lysis buffer. It consists of five main components, 50 mM Tris(pH 7.4), 150 mM NaCl, 1% NP-40, 0.5% sodium deoxycholate and 0.1% SDS, respectively. In addition, it includes many kinds of proteinase inhibitors, like sodium orthovanadate, sodium fluoride, EDTA and leupeptin, which can effectively inhibit protein degradation. PVDF membranes (Millipore Corporation, Billerica, MA, USA) were blocked in 5% non-fat dried milk and incubated overnight at 4°C with appropriate primary antibodies. The primary antibodies used were as follows: rabbit-anti-human VASH2 polyclonal antibody [11], mouse-anti-human p53 (Sigma−Aldrich, St. Louis, MO, USA), rabbit-anti-human Bax (Cell Signaling Technology, 2772, Danvers, MA, USA), rabbit-anti-human CC-3 (Beyotime, AC033, Nantong, China), and GAPDH (Beyotime, AG019-1, Nantong, China).

### Statistical analysis

All experiments were repeated in triplicate. Data were expressed as the mean ± SD. Statistical significance between two groups was determined by Student's *t*-test. The association between VASH2 expression and clinicopathological parameters was examined by the Kruskal−Wallis test. *P* values <0.05 were considered statistically significant.

## Results

### Increased VASH2 expression in HCC tissues

**Figure 1:**
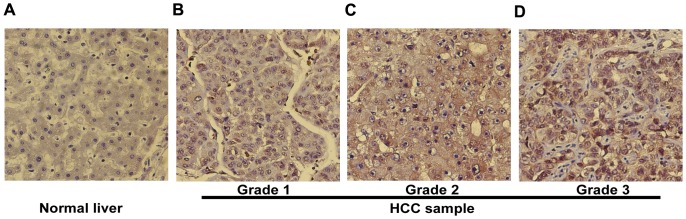
Expression of VASH2 in tissues. IHC analysis of VASH2 in human normal liver tissue (A) and human liver cancer tissue (B–D) at 20× magnification. In (A), we did not observed VASH2 protein in the cytoplasm. (B) The level of VASH2 protein localized in the cytoplasm was weakly stained. (C) The cytoplasm was moderately stained. (D) Strong staining was observed.

To confirm the expression level of VASH2 in tissues, we measured VASH2 expression in 4 normal liver tissue samples and 97 HCC tissue samples by IHC (Table 1). We observed that VASH2 expression in normal liver tissue samples was very low but was significantly higher in HCC tissues. In addition to, VASH2 expression gradually increased with decreased degree of tumor differentiation (Figure 1). Next, we examined the correlation of VASH2 expression with clinicopathological features, and the results are summarized in Table 2. High expression of VASH2 was observed in the most poorly differentiated HCC samples. In 14 cases that lacked VASH2 expression, 11 were mostly well and moderately differentiated samples. A significant correlation was observed between cancer differentiation based on Kruskal−Wallis statistical analysis (*P*<0.01), but no significant correlation was found between clinical TNM classification (*P*>0.05). Many articles have also reported that the curative effect of chemotherapeutic drug is correlated to the grade of tumor differentiation [12]–[18]. Taken together, our results showed that VASH2 was highly expressed in HCC tissues, indicating that VASH2 may be activated during tumorigenesis and plays an important role in chemotherapy.

**Table 1:** Patient Demographic Features.

Category	Subcategory	Result (%)
**Hepatocelluar carcinoma (n = 97)**		
Age	Average	49.44
	Range	28–71
Gender	Male	83(85.57)
	Female	14(14.43)
Pathological grade*	1	17(17.53)
	2	51(52.58)
	3	29(29.90)
Clinical TNM classification	I (T1N0M0)	3(3.09)
	II (T2N0M0)	28(28.87)
	IIIA(T3N0M0)	65(67.01)
	IIIC(T3N1M0)	1(1.03)
**Normal liver (n = 4)**		
Age	Average	42
	Range	38–50

*The grade 1-3 in Pathology Diagnosis is equivalent to well-differentiated, moderately-differentiated or poorly-differentiated, respectively, under microscope.

**Table 2:** Relationship between VASH2 Expression, Pathological grade and Clinical TNM classification (n = 97).

	VASH2 expression				
	0	+	++	+++	
**Pathological grade**					
1	9	4	4	0	*p*<0.01
2	2	13	19	17	
3	3	4	9	13	
**Clinical TNM classification**					
I(T1N0M0)	0	0	3	0	*p*>0.05
II(T2N0M0)	0	5	13	10	
IIIA(T3N0M0)	14	16	16	19	
IIIC(T3N1M0)	-	-	-	1	

### Generation and identification of stably transfected cells

**Figure 2:**
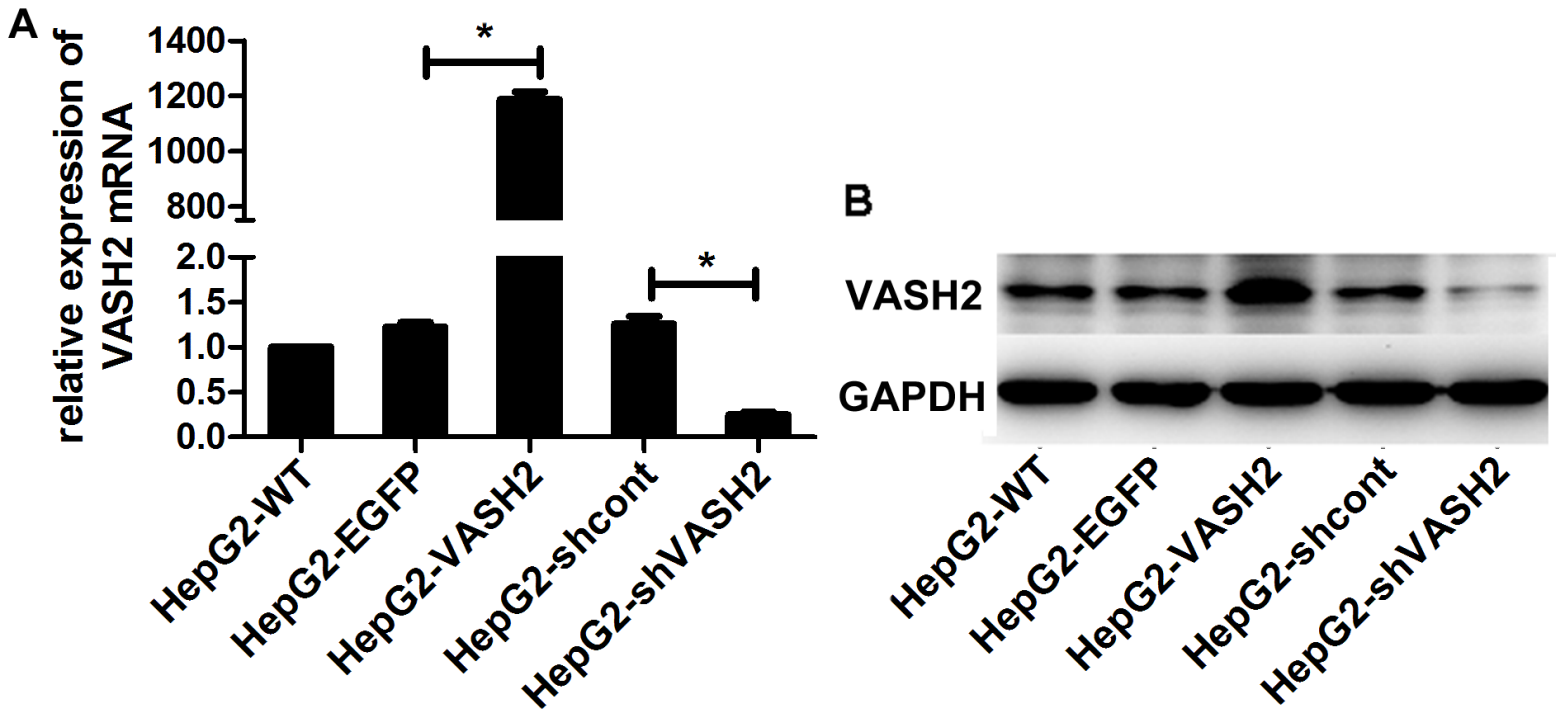
Generation and identification of stably transfected HepG2 cells. (A) Measurement of VASH2 expression using qRT-PCR. HepG2-VASH2 means VASH2-overexpressing HepG2 cells; HepG2-EGFP means HepG2 cells transfected with vector-expressing EGFP. HepG2-wt means wild-type HepG2 cells. HepG2-shcont means HepG2 cells transfected with a vector-expressing control shRNA of shVASH2. HepG2-shVASH2 means VASH2-knockdown HepG2 cells. VASH2-overexpressing HepG2 cells showed almost 1200-fold higher VASH2 expression than wild-type HepG2 cells, whereas VASH2-knockdown HepG2 cells had 80% lower expression compared with wild-type HepG2 cells (*P<0.05, compared with the control group). (B) Western blot analyses were used to confirm the knockdown efficiency. The results were similar to those of qRT-PCR analyses.

To further investigate the functions of VASH2 in HCC, we overexpressed and silenced VASH2 expression. We constructed VASH2 overexpression and VASH2-knockdown lentiviral constructs, infected HepG2 cells, and selected stably infected cells for further study. We confirmed expression levels using qRT-PCR and Western blot analysis (Figure 2). Stable cells of SMMC7721 were treated the same as HepG2 (Figure S1).

### VASH2 decreased the sensitivity of HCC cells to CDDP

**Figure 3:**
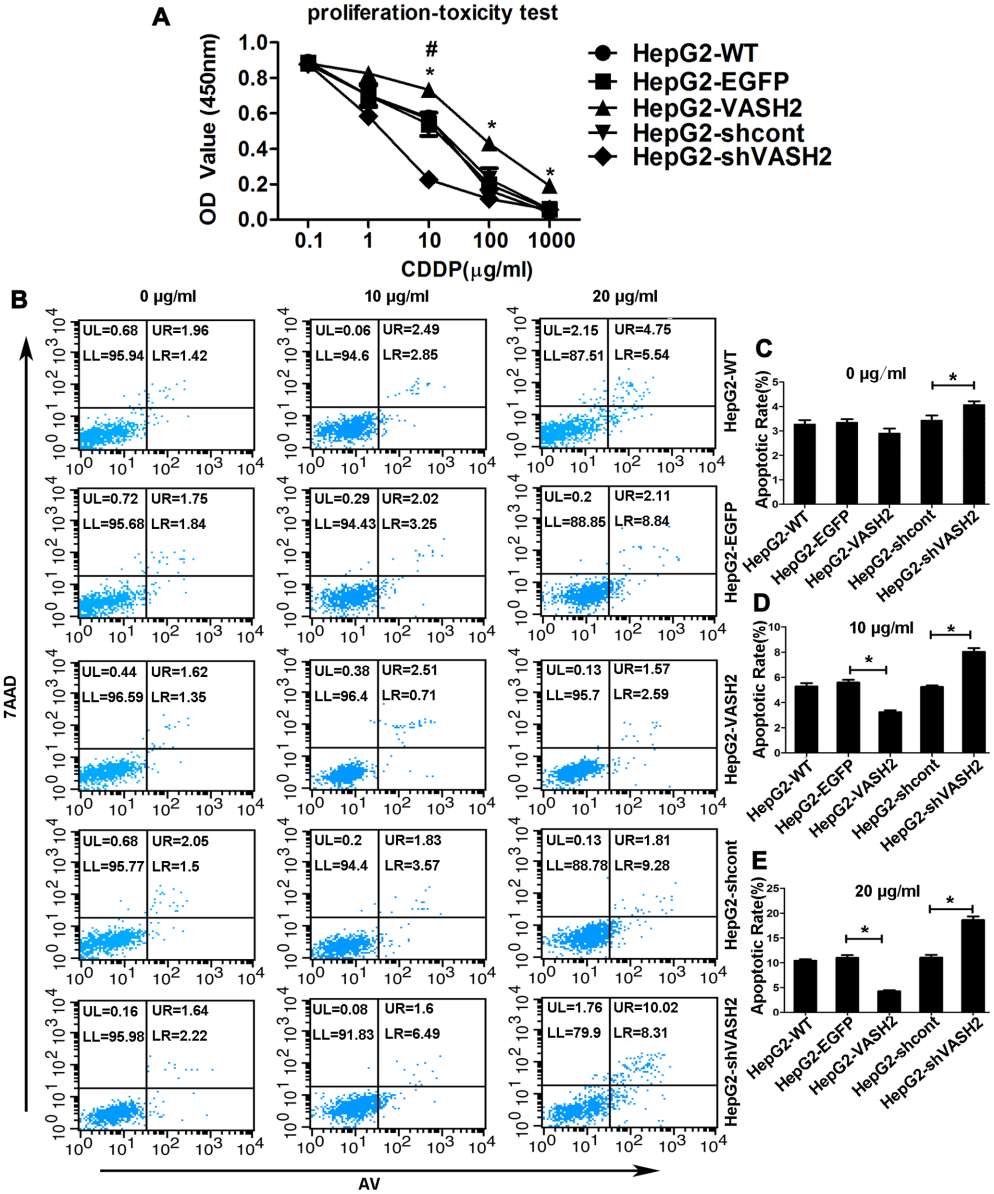
Effects of VASH2 on the sensitivity of HepG2 cells to CDDP. (A) Cell proliferation−toxicity test was conducted using Cell Counting Kit-8 (CCK-8) assay for 48 h. The overexpression of VASH2 decreased the sensitivity of CDDP (*P<0.05). By contrast, the knockdown of VASH2 increased the sensitivity of CDDP (#*P*<0.05). (B) After treatment with 0, 10 or 20 µg/ml CDDP for 48 h, the apoptosis rate was analyzed with flow cytometry. UR + LR indicated apoptosis. (C, D, and E) Quantification of the data from Figure 3B (*P<0.05).

Cytotoxicity tests were performed to study the influence of VASH2 on the cytotoxicity of HepG2 cells to CDDP. We cultured HepG2 cells with CDDP at different concentrations (1000 → 0.1 µg/ml). After incubation with CCK-8 reagent, we measured the absorbance using a microtiter plate reader. Figure 3A shows that cells expressing VASH2 presented lower toxicity of CDDP and higher cell viability than the control, whereas cells silencing VASH2 presented higher toxicity of CDDP and lower cell viability. The same results were got in SMMC7721 cells (Figure S2A).

To further assess the effect of VASH2 on the sensitivity of HepG2 cells to chemotherapy, we examined the cell apoptosis rate of HepG2 cells after treatment with 10 or 20 µg/ml CDDP for 48 h using flow cytometry. The apoptosis rate of VASH2 knockdown HepG2 cells was higher than that of the control groups (*P*<0.05). Meanwhile, VASH2 overexpression reduced the apoptosis rate compared with the control groups (*P*<0.05). VASH2 overexpression also decreased the apoptosis rate of HepG2-VASH2 cells treated with 10 µg/ml CDDP (3.24%±0.15%) compared with HepG2-EGFP cells (5.59%±0.22%) (*P*<0.05) (Figures 3B and 3D). The apoptosis rate after treatment with 20 µg/ml CDDP was 4.29%±0.21% and 10.98%±0.58% in HepG2-VASH2 and HepG2-EGFP cells, respectively (*P*<0.05) (Figures 3B and 3E). After treatment with 10 µg/ml CDDP, VASH2 knockdown increased the apoptosis rate of HepG2-shVASH2 cells treated with 10 µg/ml CDDP (8.03%±0.292%) compared with HepG2-shcont cells (5.23%±0.12%) (*P*<0.05) (Figures 3B and 3D). The apoptosis rate after treatment with 20 µg/ml CDDP was 18.61%±0.74% and 11.03%±0.58% in HepG2-shVASH2 and HepG2-shcont cells, respectively (*P*<0.05) (Figures 3B and 3E). The same results were got in SMMC7721 cells (Figure S2). These results suggested that VASH2 decreased the CDDP sensitivity of HCC cells.

### VASH2 downregulated the p53-Bax-caspase-3 pathway *in vitro*

**Figure 4:**
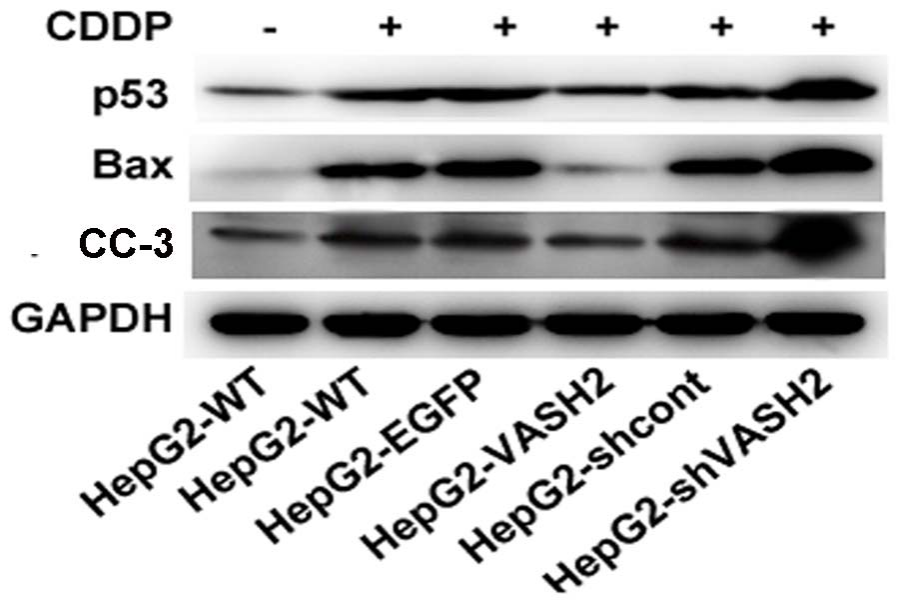
VASH2 downregulated the p53-Bax-caspase-3 pathway in HepG2 cells *in vitro*. Western blot analyses were performed to detect the protein level of p53, Bax, and CC-3. GAPDH served as a loading control.

To elucidate the mechanisms underlying VASH2 involvement in the resistance of liver cells to CDDP, we investigated the expression of several key proteins on the p53 pathway in VASH2 knockdown or overexpression HepG2 cells. Cells were treated with or without 20 µg/ml CDDP for 48 h, and then total RNA and protein were extracted, respectively. VASH2 overexpression suppressed the protein level of p53, whereas the knockdown of VASH2 resulted in enhanced protein level of p53. However, VASH2 did not influence the mRNA level of p53 (data not shown), suggesting that VASH2 down-regulated p53 at the posttranscriptional level. We also found that the proteins of Bax and CC-3, the downstream proteins of p53, decreased with VASH2 overexpression (Figure 4). The same results were got in SMMC7721 cells (Figure S3). These data indicated that VASH2 might influence the CDDP sensitivity through the p53-Bax-caspase-3 apoptotic pathway.

### VASH2 decreased the sensitivity of tumor cells to CDDP *in vivo*

**Figure 5:**
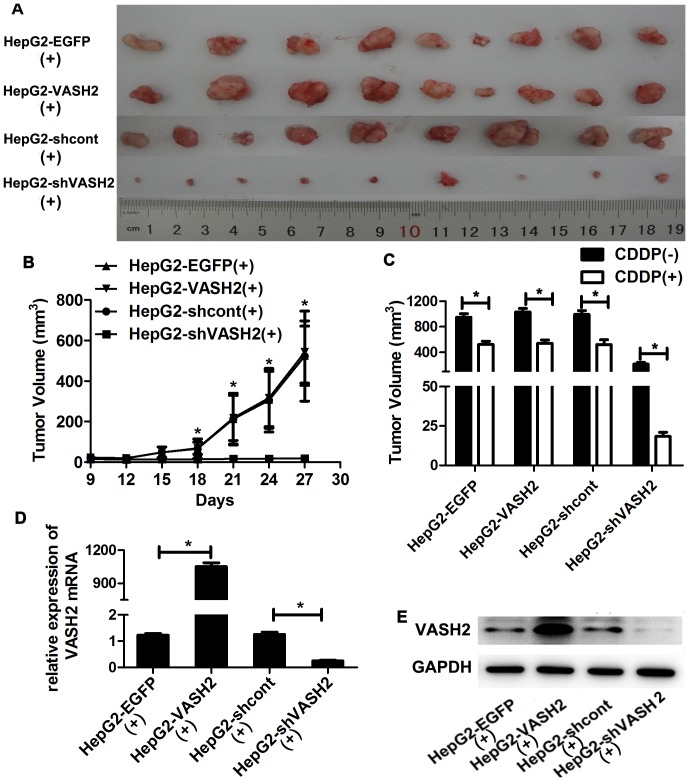
VASH2 influenced the sensitivity of tumor cells to CDDP *in vivo*. HepG2-VASH2, HepG2-EGFP, HepG2-shVASH2, or HepG2-shcont cells were suspended at a density of 10^7^ cells/ml, and 100 µl was injected into the flank of nude mice (n  =  9). On day 9, mice in CDDP(+) group were given CDDP (10 mg/kg) intraperitoneally every 3 days. After six 3-day cycles of treatment, all nude mice were sacrificed, and tumors were excised from nude mice. (A) The HepG2-shVASH2 tumors were smaller than those of the HepG2-shcont groups, whereas the size of the HepG2-VASH2 tumors did not significantly differ from that of HepG2-EGFP tumors. (B) Tumor growth curves. Tumor volume was calculated 3 days after the first treatment with CDDP using the formula (*W* ^2^×*L*)/2 every 3 days. The data are presented as the mean ± SD of nine tumors per group. *A significant difference was found between the HepG2-shVASH2 and HepG2-shcont groups (*P*<0.05). (C) The comparison of tumor volume between CDDP(+) group and CDDP(−) group(*P<0.05). (D and E) Total RNA and protein were extracted from CDDP(+) group randomly, and VASH2 expression was measured by qRT-PCR and Western blot analyses.

To further study the effects of VASH2 on the resistance of HCC to CDDP, an *in vivo* chemosensitivity experiment was performed by the subcutaneous transplantation of transduced cells into BALB/c nude mice. After injection with liver cancer cells for 9 days, mice in CDDP(+) group were intraperitoneally given CDDP every 3 days. We measured the size of the growing tumors every 3 days for 18 days, after which the mice were euthanized. The tumor sizes of the HepG2-shVASH2 group were significantly smaller than that of the HepG2-shcont, but the HepG2-VASH2 group did not show greater tumor growth than the HepG2-EGFP group (Figure 5A). The growth curves of the tumors were also generated, and we found that after 9 days of CDDP treatment, the tumors of the VASH2-knockdown group were significantly smaller (*P*<0.05) (Figure 5B). In CDDP(-) group (Figure S4), we got the similar results as the report of Xue et al [8]. Compared with CDDP(−) group, tumors were smaller in CDDP(+) group, especially knockdown of VASH2 (*P*<0.05) (Figure 5C). The tumors were excised, and RNA and protein were extracted to confirm that the stable transduction of VASH2 was maintained (Figures 5D and 5E). These results suggested that VASH2 participated in the CDDP sensitivity of HepG2 cells, in particular, the knockdown of VASH2 significantly increased CDDP toxicity.

**Figure 6:**
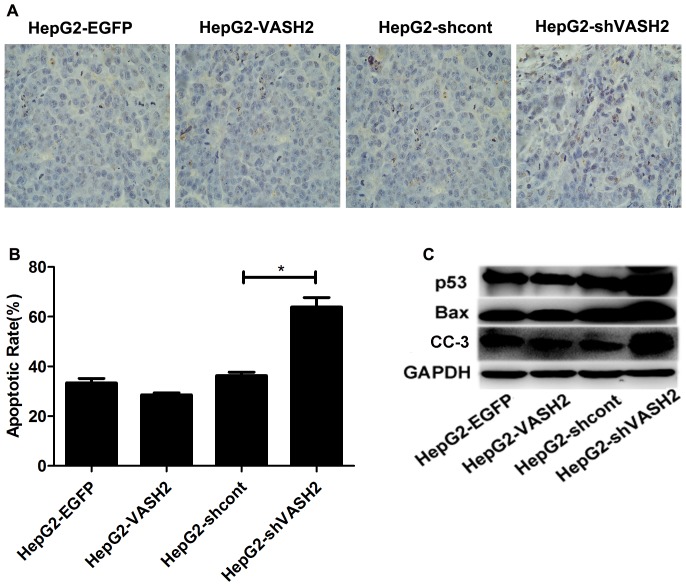
Knockdown of VASH2 enhanced the apoptosis of tumor cells by CDDP treatment. (A and B) TUNEL analysis showing enhanced apoptosis rate in tumor cells when the HepG2-shVASH2 group was compared with the HepG2-shcont group (*P*<0.05). However, no statistical difference was found in HepG2-VASH2 vs. HepG2-EGFP (*P*>0.05) at 40× magnification. (C) Western blot analysis showed that p53, Bax, and CC-3 were highly expressed in the HepG2-shVSAH2 group.

We hypothesized that the inhibition of tumor growth in the HepG2-shVASH2 group might due to increased cell apoptosis, which was confirmed by TUNEL assay on the tumor samples. Counts of apoptotic cells in each treatment group showed that the proportion of apoptotic cells in the HepG2-shVASH2 group was significantly higher than that in the HepG2-shcont group (*P*<0.05), but no difference was observed in the HepG2-VASH2 group compared with the HepG2-EGFP group (*P*>0.05) (Figures 6A and 6B).

To further explore the mechanism underlying this phenomenon, Western blot analysis was performed to measure the expression level of p53, Bax, and CC-3. The tumors from the HepG2-shVASH2 group contained a significantly higher level of p53, Bax, and CC-3 protein compared with the HepG2-shcont group, whereas no significant difference was observed in HepG2-VASH2 vs. HepG2-EGFP (Figure 6C). These data suggested that the knockdown of VASH2 significantly increased the CDDP sensitivity of tumor cells by upregulation the expression of p53, Bax and CC-3.

## Discussion

HCC is one of the most fatal diseases worldwide, and its incidence is increasing in many countries including China [30], [31]. Apart from surgical treatment, systematic chemotherapy plays an important role in HCC treatment especially for patients with advanced HCC [32]. Currently, chemotherapy is ineffective for HCC treatment because of the inherent chemoresistance. However, the exact mechanism underlying chemotherapy resistance in hepatocarcinoma is largely unknown. VASH2, firstly described by Shibuya et al. [1], has been found to promote angiogenesis [8]–[10]. Our previous studies on VASH2 have demonstrated that it is highly expressed in HCC cell lines and tissues, and it promotes HCC angiogenesis and malignant transformation by histone modification [8]. Interestingly, we discovered in the present study that, in addition to high VASH2 expression in tissues, the protein level of VASH2 gradually increased with decreased degree of tumor differentiation (*P*<0.01), which is reportedly associated with the curative effect of chemotherapeutic drugs [12]–[18]. However, no relationship was found between VASH2 expression and clinical TNM classification (*P*>0.05). Based on these data, we hypothesized that VASH2 may be involved in the mechanism of cancer chemotherapy.

To confirm this hypothesis, we successfully constructed VASH2 overexpression and knockdown cell lines. We found that overexpressed VASH2 can decrease the toxicity and rate of cell apoptosis (*P*<0.05), especially the knockdown of basal VASH2 expression by cell proliferation−toxicity and flow cytometry tests after CDDP exposure. However, *in vivo* chemosensitivity study showed no difference between overexpressed VASH2 and HepG2-EGFP groups (*P*>0.05). Two reasons can explain this phenomenon. First, HepG2-EGFP cells already had relatively high endogenous VASH2 expression. Second, due to the influence of drug absorption and degradation and the insufficient of blood supply to implanted tumors, the concentration of CDDP is less than 10 mg/kg, so no difference between overexpressed VASH2 and HepG2-EGFP groups is got, but significant difference between knockdown VASH2 and HepG2-shcont groups.

CDDP is a common chemotherapeutic agent used for HCC. Patients usually have a good initial response to CDDP-based chemotherapy but later relapse because CDDP resistance develops in either acquired or intrinsic form, thereby markedly reducing the clinical effectiveness of this drug [20]. Some articles have reported that many mechanisms underlie the resistance of CDDP, such as nucleotide excision repair (NER) system and DNA mismatch repair (MMR) system [33], [34]. However, clinical tests show unsatisfactory results for HCC treatment. Therefore, novel pathways must be identified for HCC patients who are resistant to chemotherapy. VASH2, a member of the VASH family, promotes angiogenesis. Moreover, VASH2 overexpression stimulates cell proliferation [8]. One of the mechanisms underlying CDDP resistance is the inhibition of cell apoptosis through the increase in p53 protein levels. *In vivo* and *in vitro* assays revealed that VASH2 decreased the rate of cell apoptosis after treatment with CDDP. Thus, VASH2 may be related to p53 in cell apoptosis.

To explore the mechanisms underlying this phenomenon, we detected the level of p53, Bax, and CC-3 expression. As expected, the protein levels of p53, Bax, and CC-3 were deregulated when VASH2 was overexpressed by Western blot analysis. These results suggested that VASH2 may inhibit cell apoptosis by suppressing the p53 pathway. However, the mRNA level of p53 was not reduced (data not shown), indicating that VASH2 may deregulate p53 through post-transcriptional control, like phosphorylation or ubiquitination, which can influence the stability p53 protein [35]–[39]. But how VASH2 deregulate p53 remains to be determined. In this study, we justly selected the wild-type p53 cell lines, HepG2 and SMMC7721, to explore the function of VASH2 in chemoresistance. In about half of human carcinomas, wild-type p53 is mutated at the gene level [40], which is an important cause of drug resistance. In HCC, the worldwide prevalence of TP53 mutations has been estimated to be around 28% [41] and 37% in the Chinese population [42]. The highest rates are observed in aflatoxin-exposed populations in which >50% have a specific mutation at codon 249 [43]. Thus, the mechanism between VASH2 and the mutated type p53 needs further study.

VASH2 is reportedly expressed in many common tumors, like hepatocellular carcinoma, serous ovarian adenocarcinoma and gastric cancer, and participates in the program of tumor metabolism, such as angiogenesis [8], [10], [44]. Unlike VEGF and other angiogenic factors, it has been identified as an extrinsic and VEGF-independent angiogenic factor that is highly expressed in bone marrow-derived mononuclear cells but weakly expressed in endothelial cells [9]. In the previous study, we demonstrated that VASH2 contributed to the angiogenesis in HCC via an SVBP-mediated paracrine mechanism. And, in the present study, we also confirmed that VASH2 significantly correlated with differentiation of HCC samples and involved in the resistance of HCC cell lines to CDDP by regulating p53. This observation would give us a new insight into the biological activities of VASH2 in tumors. So, these results strongly indicate that VASH2 may be a novel target for cancer therapy and has a certain guiding role for the establishment of chemotherapy regimens. In this study, we found that VASH2 could influence the chemosensitivity in hepatocarcinoma cell lines. However, we did not have clinical data about the correlation between VASH2 expression and chemosensitivity of HCC samples. So, it is needed to further verify in clinical specimens.

## Supporting Information

Figure S1 **Generation and identification of stably transfected SMMC7721 cells.** (A) Measurement of VASH2 expression using qRT-PCR (*P<0.05, compared with the control group). (B) Western blot analyses were used to confirm the knockdown efficiency.
(TIF)

Figure S2 **Effects of VASH2 on the sensitivity of SMMC7721 cells to CDDP.** (A) Cell proliferation−toxicity test was conducted using Cell Counting Kit-8 (CCK-8) assay for 48 h. The overexpression of VASH2 decreased the sensitivity of CDDP (*P<0.05). By contrast, the knockdown of VASH2 increased the sensitivity of CDDP (#*P*<0.05). (B) After treatment with 0, 10 or 20 µg/ml CDDP for 48 h, the apoptosis rate was analyzed with flow cytometry. UR + LR indicated apoptosis. (C, D and E) Quantification of the data from Figure 3B (*P<0.05).
(TIF)

Figure S3 **VASH2 downregulated the p53-Bax-caspase-3 pathway in SMMC7721 cells** ***in vitro*** **.** Western blot analyses were performed to detect the protein level of p53, Bax, and CC-3. GAPDH served as a loading control.
(TIF)

Figure S4 **Subcutaneous injection of tumor cells.** (A) The HepG2-shVASH2 tumors were smaller than those of the HepG2-shcont groups, whereas the size of the HepG2-VASH2 tumors did not significantly differ from that of HepG2-EGFP tumors. (B) Tumor growth curves. Tumor volume was calculated using the formula (*W* ^2^×*L*)/2 every 3 days. The data are presented as the mean ± SD of 9 tumors per group. *A significant difference between the HepG2-shVASH2 and HepG2-shcont groups was found after 15 days (*P*<0.05). (C and D) Total RNA and protein were extracted from CDDP(+) group randomly, and VASH2 expression was measured by qRT-PCR and Western blot analyses.
(TIF)
