# Supplementary figures and images for: Vasohibin 2 Decreases the Cisplatin Sensitivity of Hepatocarcinoma Cell Line by Downregulating p53
Source: PLoS One. 2014 Mar 4;9(3):e90358. doi: 10.1371/journal.pone.0090358 (PMC3942424; doi:10.1371/journal.pone.0090358)

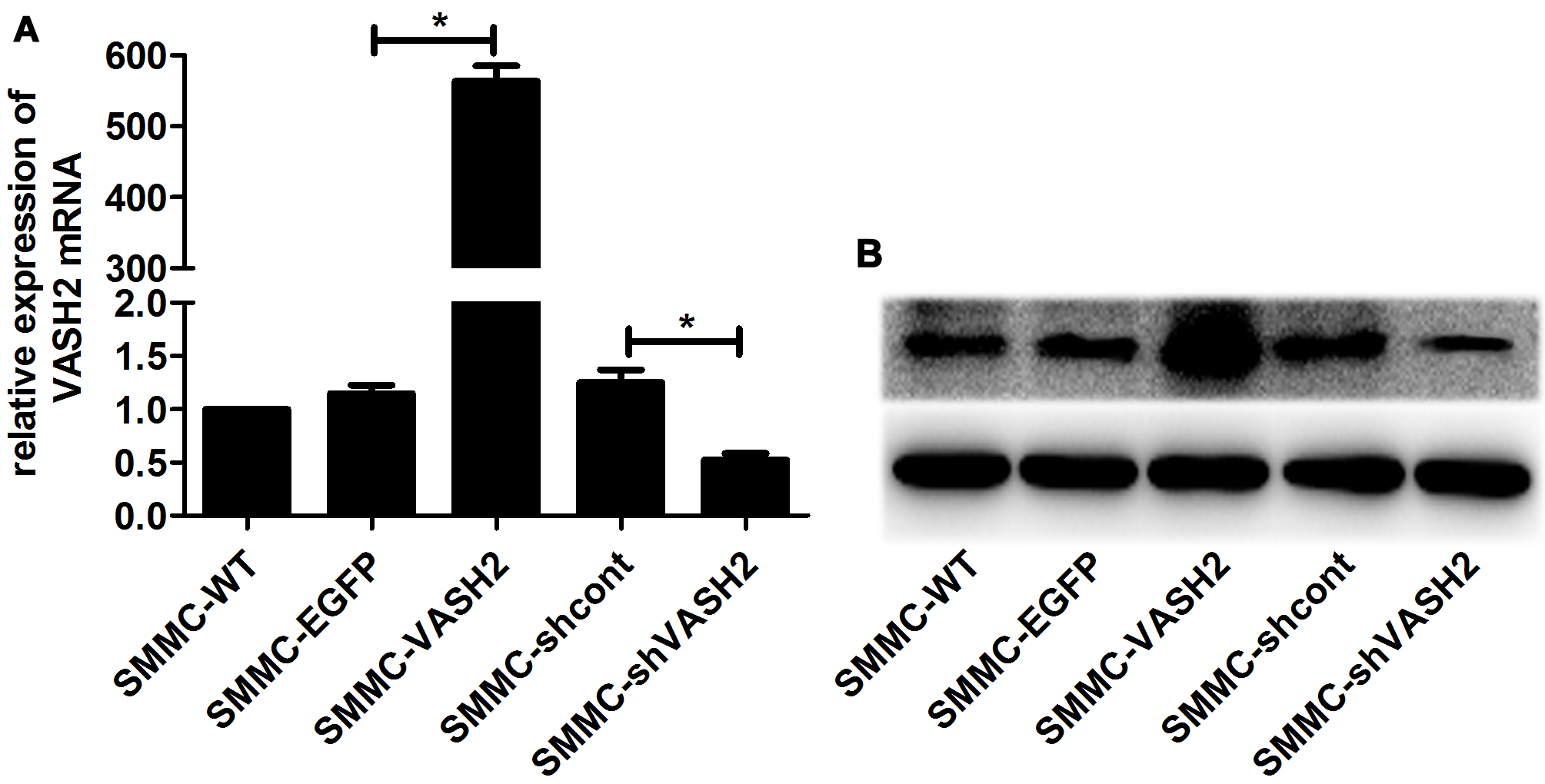

Supplement: Figure S1 — Generation and identification of stably transfected SMMC7721 cells. (A) Measurement of VASH2 expression using qRT-PCR (*P<0.05, compared with the control group). (B) Western blot analyses were used to confirm the knockdown efficiency. (TIF) [file pone.0090358.s001.tif]

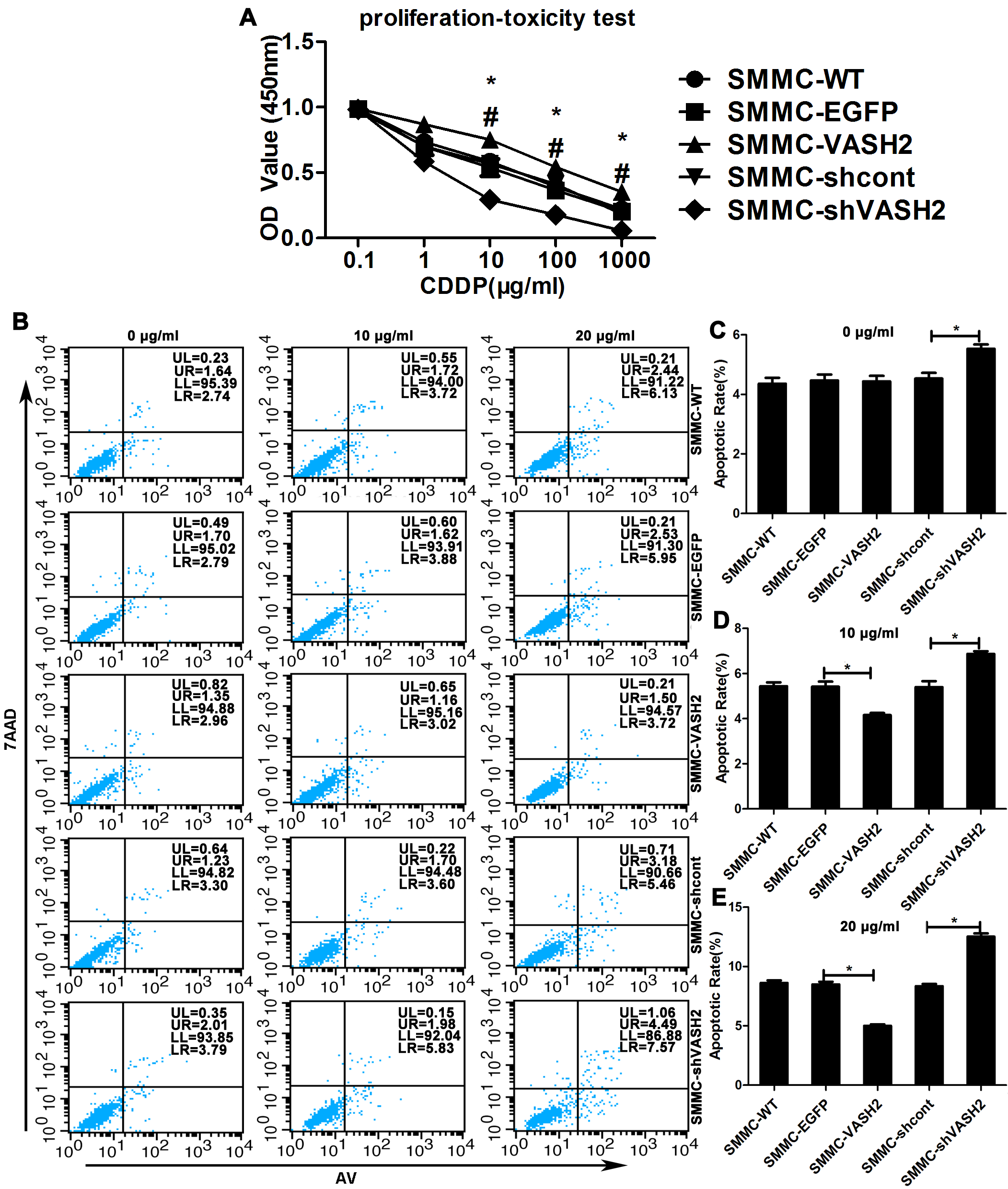

Supplement: Figure S2 — Effects of VASH2 on the sensitivity of SMMC7721 cells to CDDP. (A) Cell proliferation−toxicity test was conducted using Cell Counting Kit-8 (CCK-8) assay for 48 h. The overexpression of VASH2 decreased the sensitivity of CDDP (*P<0.05). By contrast, the knockdown of VASH2 increased the sensitivity of CDDP (#P<0.05). (B) After treatment with 0, 10 or 20 µg/ml CDDP for 48 h, the apoptosis rate was analyzed with flow cytometry. UR + LR indicated apoptosis. (C, D and E) Quantification of the data from Figure 3B (*P<0.05). (TIF) [file pone.0090358.s002.tif]

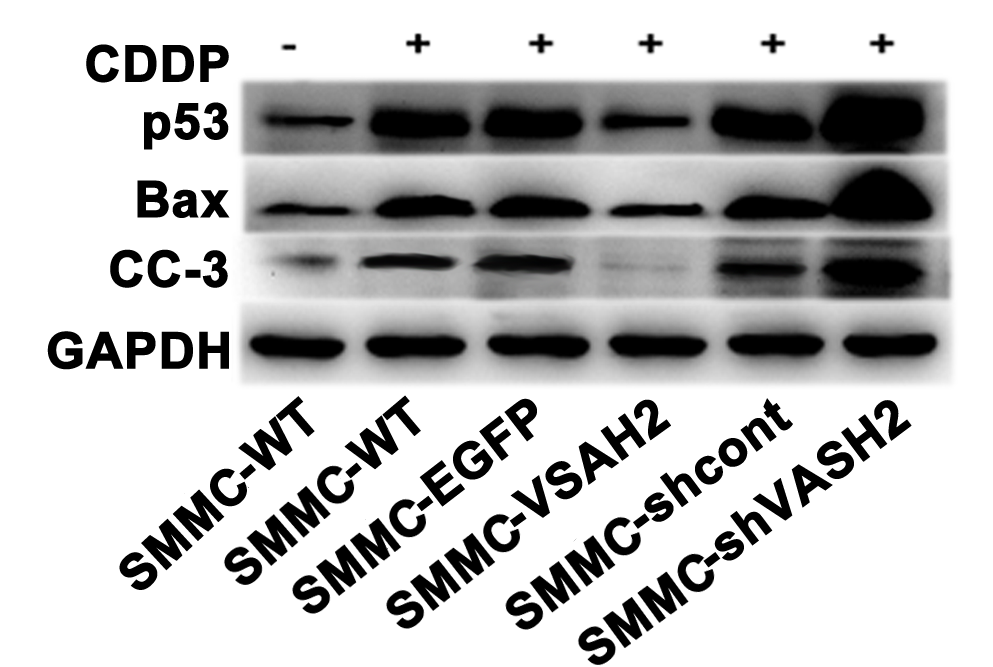

Supplement: Figure S3 — VASH2 downregulated the p53-Bax-caspase-3 pathway in SMMC7721 cells in vitro . Western blot analyses were performed to detect the protein level of p53, Bax, and CC-3. GAPDH served as a loading control. (TIF) [file pone.0090358.s003.tif]

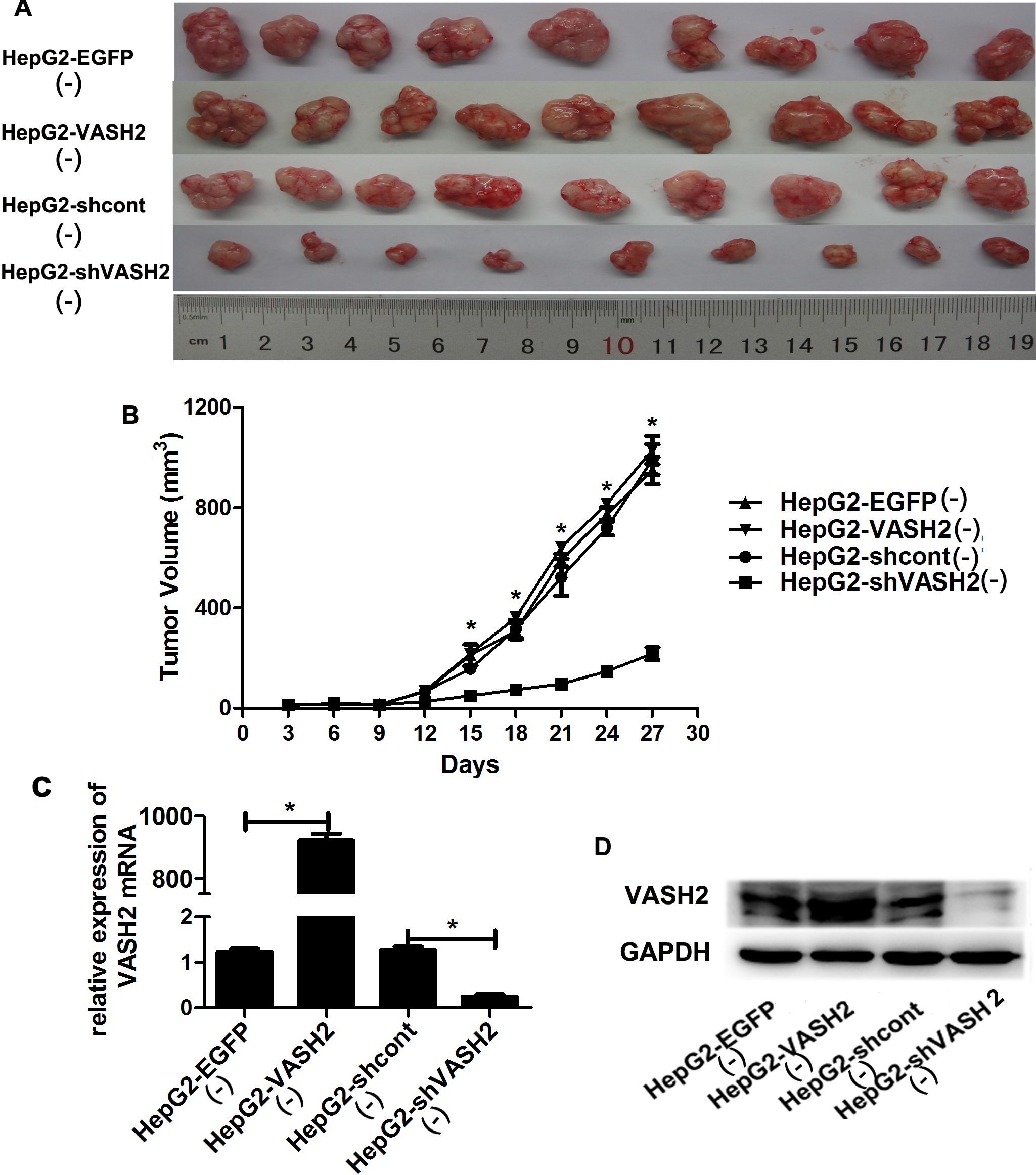

Supplement: Figure S4 — Subcutaneous injection of tumor cells. (A) The HepG2-shVASH2 tumors were smaller than those of the HepG2-shcont groups, whereas the size of the HepG2-VASH2 tumors did not significantly differ from that of HepG2-EGFP tumors. (B) Tumor growth curves. Tumor volume was calculated using the formula (W 2×L)/2 every 3 days. The data are presented as the mean ± SD of 9 tumors per group. *A significant difference between the HepG2-shVASH2 and HepG2-shcont groups was found after 15 days (P<0.05). (C and D) Total RNA and protein were extracted from CDDP(+) group randomly, and VASH2 expression was measured by qRT-PCR and Western blot analyses. (TIF) [file pone.0090358.s004.tif]
